# SARS-CoV-2 Spike-Mediated Entry and Its Regulation by Host Innate Immunity

**DOI:** 10.3390/v15030639

**Published:** 2023-02-27

**Authors:** Shi Yu, Huina Hu, Qiangyun Ai, Rong Bai, Kaixiong Ma, Minmin Zhou, Shaobo Wang

**Affiliations:** 1 State Key Laboratory of Respiratory Disease, Guangzhou Medical University, Guangzhou 511400, China; 2 Guangzhou Laboratory, Department of Basic Research, Guangzhou International Bio-Island, Guangzhou 510005, China

**Keywords:** SARS-CoV-2, spike, S1, S2, host proteases, proteolytic processing, virus host interaction, innate immune responses

## Abstract

The constantly evolving severe acute respiratory syndrome coronavirus 2 (SARS-CoV-2) variants of concern (VOC) fuel the worldwide coronavirus disease (COVID-19) pandemic. The spike protein is essential for the SARS-CoV-2 viral entry and thus has been extensively targeted by therapeutic antibodies. However, mutations along the spike in SARS-CoV-2 VOC and Omicron subvariants have caused more rapid spread and strong antigenic drifts, rendering most of the current antibodies ineffective. Hence, understanding and targeting the molecular mechanism of spike activation is of great interest in curbing the spread and development of new therapeutic approaches. In this review, we summarize the conserved features of spike-mediated viral entry in various SARS-CoV-2 VOC and highlight the converging proteolytic processes involved in priming and activating the spike. We also summarize the roles of innate immune factors in preventing spike-driven membrane fusion and provide outlines for the identification of novel therapeutics against coronavirus infections.

## 1. Overview of the SARS-CoV-2 Virus

Severe acute respiratory syndrome coronavirus 2 (SARS-CoV-2) is the causative agent of the COVID-19 pandemic. The initial case of SARS-CoV-2 was reported in Wuhan 2019, and reported to be a zoonotic spill-over event, possibly from bats to the human population [1,2,3], followed by rapid community transmission that later developed into a global pandemic. Among many modes of transmission, SARS-CoV-2 transmits through air and infects the human respiratory cells to cause acute respiratory distress syndrome (ARDS) and severe pneumonia, which have been the leading causes of death [4,5]. SARS-CoV-2 transmits more swiftly than SARS-CoV, or the middle east respiratory coronavirus (MERS-CoV), making it the most challenging human coronavirus to date. Although scores of SARS-CoV-2 vaccines have been developed and approved worldwide, vaccines only provide limited effectiveness in curbing the SARS-CoV-2 transmission [6,7,8]; in addition, emerging SARS-CoV-2 variants of concern (VOC) and Omicron subvariants are extending the global spread, posing a continuing threat to vaccine efficacy [9]. Understanding the biology of SARS-CoV-2 entry and human host defenses is of great research priority for therapeutic approaches and future coronavirus emergence.

**Figure 1:**
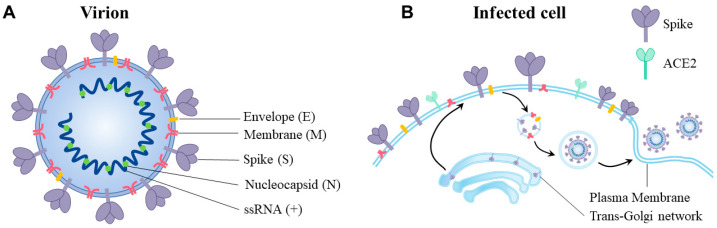
Display of SARS-CoV-2 proteins on virions and infected cells. (**A**) SARS-CoV-2 virions are enveloped spherical particles composed of four major structural proteins, namely the spike (S), membrane (M), envelope (E), and nucleocapsid (N). In situ virions were 90 ± 10 nanometers in diameter, while the positive-sense RNA genome is encapsulated in the double membrane derived from host cells and is to be released into host cell cytoplasm upon viral entry. (**B**) Apart from free virions, host cells infected with SARS-CoV-2 also display viral structural proteins in distinct subcellular compartments, mainly the plasma membrane, intracellular organelles, and cytoplasm. Replication and assembly near the trans-Golgi network occur before the release of viral particles to the extracellular environment.

SARS-CoV-2 is an enveloped, positive-sense, single-stranded RNA virus. Its genome encodes four structural proteins including the spike (S), envelope (E), nucleocapsid (N), and membrane (M) (Figure 1A). When assembled onto virions, these structural proteins stabilize the approximate ~30-kilobase viral genome within the viral envelope and support the delivery of viral RNA into the host cell cytoplasm, in order to initiate the subsequent viral replication. SARS-CoV-2 free virions exist at high concentrations in aerosols and fomites, which travel short distances in the air or last on the contaminated objects, until free virions are inhaled deeply into the human airway [5,10,11].

Upon contacting the target cell, spike proteins on the SARS-CoV-2 virions attach to the angiotensin-converting enzyme 2 (ACE2) to enter the cell [12]. ACE2 is highly expressed in the epithelial linings of the upper respiratory tract, namely the nasal cavity [13,14,15], where these tissues efficiently support air transmission through droplets. Notably, the ACE2 receptor is highly expressed in other human organs, such as the digestive tract, heart, kidney, and reproductive tissues [15]. The SARS-CoV-2 entry process is primarily driven by the structural protein spike, where the binding of ACE2 facilitates its proteolytic activation by host proteases. The proteolytically cleaved spikes rearrange themselves to induce the fusion of viral and host membranes at the cell surface or inside the endosomal compartment. 

Having once entered the target cells, the viral RNA genome is rapidly translated into effector molecules that play vital roles in supporting the viral replication cycle. Structural proteins, such as spikes, M, and E, undergo complex trafficking events for their maturation before being displayed onto the plasma membrane or assembled onto virions [16,17] (Figure 1B). Non-structural proteins (NSPs) in the cytoplasm, such as NSP7, NSP8, NSP12, and NSP13, form the RNA-dependent RNA polymerase (RdRp) complex for the synthesis of the viral genome [18,19,20,21]. While viral proteases that promote the maturation of viral components from polyproteins, such as the NSP3 and NSP5 (also known as the papain-like protease, PLpro, and the main protease, Mpro, respectively) [22,23], can also cleave host factors to evade innate immune responses in target cells [24,25,26], other effectors targeting membrane organelles disrupt cellular homeostatic processes to facilitate viral replication. For instance, Orf9b specifically targets the mitochondrial membrane TOM70 and suppresses the mitochondrial antiviral signaling protein (MAVS) [27,28]. 

Viral entry is the initial and essential step in the infection cycle. Hence, it is important to understand the mechanism of SARS-CoV-2 entry, either by the ancestral strain or the ongoing Omicron subvariants. Molecular events targeting the spike not only limit the initial SARS-CoV-2 viral entry, but also inhibit downstream viral replication to reduce potential cellular damage and host pathology.

## 2. The SARS-CoV-2 Spike

Among all SARS-CoV-2 encoded proteins, spikes are responsible for the initial host receptor recognition, membrane fusion, and virion assembly. Similar to the influenza A virus (IAV) hemagglutinin (HA), human immunodeficiency virus (HIV) envelope, the SARS-CoV-2 spike glycoprotein is a class I fusion protein that requires host receptor binding to initiate its activation [29]. Upon binding to the target cell receptor, the spikes undergo a major conformational change, which then allows a proteolytic cleavage event via the target cell proteases to unlock an intermediate structure that tethers the viral and cell membrane together [30]. Eventually, spike-mediated membrane fusion is essential for the subsequent delivery of the viral RNA genome and viral replication in the infected host cell. Hence, the spike proteins displayed on the viral and cell membranes have been a primary target of current therapeutics.

The ancestral strain SARS-CoV-2 spike protomer has 1273 amino acids and is expressed and inserted into the endoplasmic reticulum (ER)membrane, before being trafficked through the Golgi network for a complex series of post-translational modifications, including adding 22 N-linked glycosylation and C-terminal palmitoylation [29,31,32]. Optimally folded and matured spikes form homotrimers before being trafficked to the plasma membrane or assembled onto the virions as pre-fusion spike trimers [29]. SARS-CoV-2 spikes carry a polybasic S1/S2 junction that can be cleaved into the amino-terminal (N-terminal) S1 and carboxy-terminal (C-terminal) S2 subunits via host cell furin during its maturation [12,33]. Most of the spike structural works carried out were using secreted and prefusion forms of spikes (in the absence of a transmembrane domain), showing S1 subunits forming the outer shells of the spike trimer and wrapping around the more densely folded S2 subunits [32,34]. The pre-fusion S2 hinge is flexible, and spike trimers can display angled and tilted conformations on the SARS-CoV-2 virions [35,36,37].

The physical number of spikes residing on virions may directly influence the strength of viral attachment, infectivity, and immune antigenicity. In situ cryo-electron tomography of the ancestral SARS-CoV-2 virions revealed that each spherical viral particle has a diameter of 85 ± 10 nanometers and is estimated to carry 30–40 spike homotrimers [35,36,37,38] predominantly presenting in their pre-fusion states. When compared to SARS-CoV, the wild-type SARS-CoV-2 virions carry approximately 30% less spike trimers [39,40], and it is unclear whether the number of spikes per virion differs between other SARS-CoV-2 VOC.

**Figure 2:**
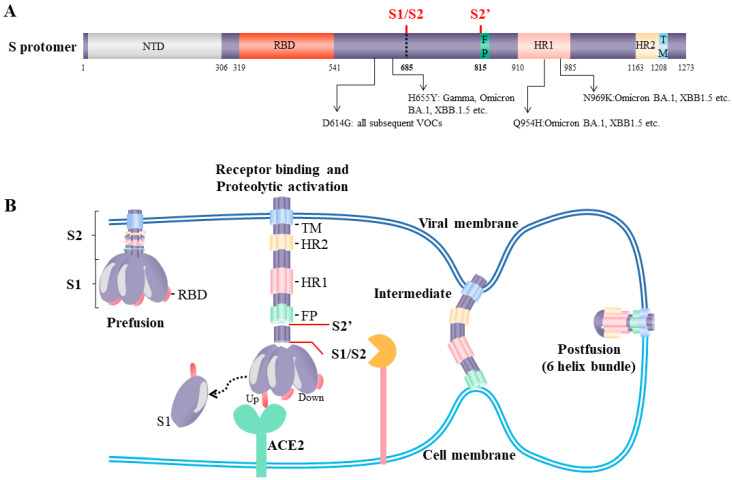
(**A**) Schematics of SARS-CoV-2 full-length spike protomer, selected domains, proteolytic sites and converging mutations in variants of concern (VOC); (**B**) the proposed model of spike-mediated membrane fusion. SARS-CoV-2 spike homotrimer recognizes target cell ACE2, followed by proteolytic processing at the S1/S2 and S2′ sites to initiate a conformational rearrangement within the S2. The S2′ cleavage enables the release of fusion peptide (FP) that anchors the target cell membrane. Subsequently, the S2′ structural transition allows the formation of coiled–coil interactions between the HR1 and HR2 domains. This interaction ultimately drives the fusion of viral and host membranes, enabling the release of the viral genome into the infected cells.

The SARS-CoV-2 S1 subunit contains a receptor-binding domain (RBD) (Figure 2A). The RBD recognizes human angiotensin-converting enzyme 2 (ACE2) [41], a functional receptor originally identified for SARS-CoV [42], and human coronavirus (HCoV-NL63) [43]. Point mutations occurring within the spike RBD can further increase ACE2-binding affinity via the formation of additional hydrogen bridges or hydrophobic interactions [44,45,46,47]. Structural analysis revealed that the RBD could possess up and down configurations, in which an up-conformation favors the interaction with ACE2 or ACE2-blocking neutralization antibodies [32,41,48,49,50,51], while the RBD in down-conformation masks ACE2-interacting interfaces and hides potential antigenic epitopes [52] (Figure 2B). The configurations of the RBD can be additionally regulated by glycans, fatty acids, and physical temperature [52,53,54], or individual mutations within the S1 subunits, such as amino acid substitution at the D614G [55,56] or altered hydrophobicity at S375F [57]. Nevertheless, the S1 subunit senses the entry signal derived from target cell receptor binding and translates it into the activating signal for the S2 subunit.

The S2 subunit contains highly conserved regions that catalyze the membrane fusion. The internal fusion peptide is a highly hydrophobic membrane-interacting region [58,59] that is only exposed and functional upon sequential receptor engagement and proteolytic activation (discussed in the later section) (Figure 2A) [60,61]. The released fusion peptide interacts with target cell plasma or endosomal membranes in a calcium-dependent manner [58,62,63] in order to anchor the viral membrane to the target cell. To complete the fusion process, S2 undergoes a major structural reconfiguration, primarily driven by the rearrangement of the heptad repeat 1 (HR1) domain [64]. During the fusion process, the four α-helices in the HR1 domain extend into a single continuous long alpha helix [64] that stabilizes the C-terminal HR2 domain via a coiled–coil interaction, ultimately forming the core of the post-fusion S2 trimers (Figure 2B) [34]. Since the structural rearrangement within S2 catalyzes the fusion of tethered viral and cell membranes, it is extremely important when and where the transition of the S2 subunit occurs. 

Over the period of the COVID-19 pandemic, the spike has accumulated a variety of mutations along the S1 and S2 subunits, causing SARS-CoV-2 VOC to partially evade approved vaccines and population immunity. However, the steps involved in the priming and activation of the SARS-CoV-2 spikes remain relatively conserved. The host-mediated proteolysis of spikes could be a promising antiviral target. Yet few protease inhibitors or neutralization antibodies are available to block the activation of the spikes. Here, we emphasize the two proteolytic steps involved in spike priming and activation. 

## 3. Proteolytic Priming of the SARS-CoV-2 Spike S1 and S2 Subunits

Initial amino acid sequence alignments of the original SARS-CoV and SARS-CoV-2 spike show the addition of a polybasic cleavage site at the S1/S2 junction [12,33]. Indeed, SARS-CoV-2 spikes are proteolytically processed by host pro-protein convertase furin during its biosynthesis [29], and is similar to the spikes of the MERS-CoV and murine hepatitis virus (MHV) [65,66,67]. As a result, spikes assembled on the authentic virions or pseudotyped particles display cleaved forms of spike S1 and S2 subunits [35]. Although spikes are auto-processed during biosynthesis, S1 remains non-covalently attached to the S2 subunit; furin-cleaved spikes on cells and virions remain fusion-competent and do not display a post-fusion or activated conformation [29]. Genetic knockdown and pharmacological inhibition of furin effectively abolish the spike processing at S1/S2 [12,68], but is not essential for spike-mediated viral entry [69], suggesting that cleavage at the S1/S2 site alone does not drive the membrane fusion of the spikes. In contrast to the furin-cleaved SARS-CoV-2 spikes, the original SARS-CoV full-length spikes carry a monobasic S1/S2 cleavage site and can be also primed into S1 and S2 subunits by cells either expressing TMPRSS2 or TMPRSS11D [70,71]. 

One of the functional outcomes of the S1/S2 cleaved SARS-CoV-2 spikes has been described in terms of syncytium formation [33,68,72,73], where a SARS-CoV-2-infected cell can directly fuse with an adjacent cell expressing the receptor ACE2. Cells transiently expressing wild-type spikes alone, but not furin-cleavage-site-deficient spikes, were sufficient to drive cell–cell fusion in the absence of authentic SARS-CoV-2 infection [33,74]. SARS-CoV-2 spike-mediated syncytia formation is expected to be highly cytopathic and has been identified as a clinical hallmark driving the lung pathogenesis in humans [75,76,77]. Residues adjacent to the furin cleavage site also actively acquired additional mutations to modulate the extent of syncytia formation, including the P681H in the Alpha variant and all current Omicron subvariants, as well as the P681R in highly pathogenic Delta and certain lineage A variants [78]. A single substitution at P681H and P681R renders the S1/S2 cleavage site highly susceptible to additional proteases, or increased proteolytic processing by furin [79], therefore accelerating the spike-mediated membrane fusion and syncytium formation [80]. In addition to forming multinucleated cells, syncytia may serve a purpose for the intercellular transmission of SARS-CoV-2 components [74,81], or help to evade host innate and adaptive immune responses. Once the spikes are cleaved into S1 and S2, the polybasic C-terminus of the S1 subunit can be exposed to engage attachment receptors such as neuropilin-1 (NRP1) [82,83,84]. NRP1 binding to wildtypes, but not furin-cleavage site deficient, SARS-CoV-2 pseudotypes facilitate the viral attachment and infection of human lung epithelial cells [82,83]. Hence, the furin cleavage site of spikes promotes SARS-CoV-2 pathogenesis and transmission.

Although S1/S2 cleaved spikes are primed for membrane fusion, this cleavage also penalizes the overall stability of spike trimer. Spontaneous shedding of the S1 subunit has been reported in the absence of host receptor binding [34], and can be induced by chemical inactivation of the SARS-CoV-2 virions [85]. Soluble ACE2 and antibodies can induce the S1 shedding to prevent subsequent interaction of virion spikes and the ACE2 receptor on the target cells [86,87], rendering the spike susceptible to neutralization. A predominant mutation that occurred on all SARS-CoV-2 VOC is the spike D614G substitution. The D614G substitution increases viral infectivity by stabilizing the cleaved S1 and S2 subunits, thus increasing the S1 incorporation onto the virions and favoring the action of ACE2 binding [88,89]. In addition to increased S1 incorporation onto virions, the D614G also increased the RBD up-conformation in structurally resolved spike trimers, thus increasing the ACE2-binding affinity [56,89,90,91,92]. 

Prevalence of the spike H665Y substitution within the S1 subunit has been ranked highest among all SARS-CoV-2 VOC and subsequent Omicron subvariants [93,94,95], suggesting that H655Y plays a critical role in promoting the SARS-CoV-2 viral fitness [96]. Spike H655Y substitution in the Omicron subvariants has been shown to be responsible for the endocytic route of entry (Table 1) [94,97,98,99]. Mechanistically, H665Y improves the association of the S1 and S2 subunits and further stabilizes the spike trimer [100]. In vitro syncytia assays using cells expressing H655Y spikes showed a reduced level of syncytia, and may be contributed by the reduced opening of the S1 subunit and altered the kinetics of spike activation at the plasma membrane [100]. Thus, the exact mechanisms for H655Y altered the protease usage and endocytic entry warrants further investigation. Omicron and subvariants, including the latest BQ1.1 and XBB.1.5, all carry Q954H and N969K substitutions at the end of the HR1 domain within the S2 subunit. Spikes that carry Q954H and N969K substitutions display reduced S1/S2 cleavage in various cell lines and result in decreased VSV pseudotype entry in Caco-2 cells [101]; these substitutions did not affect the rate of syncytia formation. Q954H and N969K substitutions had no structural impact on post-fusion spikes [102], nor affecting peptide fusion inhibitors binding to the HR1 domain [103]. Hence, the mechanism of spikes that carry Q954H and N969K substitutions remains to be identified. 

## 4. Spike Proteolytic Activation and Utilization of Host Proteases

Activation of the spike-mediated membrane fusion involves the proteolytic processing at the spike S2′ cleavage site [104]. Unlike the constantly evolving spike S1/S2 cleavage site, the S2′ cleavage site remains highly conserved among all VOC and other coronaviruses. Our previous work supports that the cleavage of S2′ does not occur in the absence of host cell receptor binding, demonstrated using cell–cell fusion and pseudotype entry models in the absence or presence of host cell receptor ACE2 [61]. Furthermore, only receptor ACE2 binding triggered the formation of the proteinase K-resistant, post-fusion S2′ structure resembling the rigid six-helix bundle [61]. In SARS-CoV-2-permissive cell types that endogenously express ACE2, such as Vero E6 cells, spike expression alone is sufficient in driving the syncytium formation [33,68]. Moreover, the presence of the S2′ cleavage product has been observed in various cell types infected with authentic wild-type and furin-deficient SARS-CoV-2 viruses, regardless of the presence of the furin cleavage site [35,105]. Since S2′ cleavage strictly occurs during virus-to-cell and cell-to-cell membrane fusion, subcellular localization of this cleavage would dictate the mode of spike activation and viral entry at the plasma membrane or endosomes.

TMPRSS2 belongs to the family of type 2 transmembrane serine proteases (TTSPs) and is involved in the entry of many pathogenic viruses [106,107,108,109]. It has been shown that TMPRSS2 activates SARS-CoV-2 as well as SARS-CoV and other coronavirus spikes in the human upper and lower respiratory tract [33,71,110]. TMPRSS2 not only cleaves spikes at the S1/S2 junction [71], but also processes at the S2′ cleavage site to drive spike-mediated membrane fusion in ACE2-expressing target cells [33,107,111]. For the ancestral SARS-CoV-2 strain, the usage of TMPRSS2 was prioritized, and the entry of human lung epithelial cells do not utilize the endosomal mode of entry [109,112,113,114]; as a result, increasing endosomal pH with hydroxychloroquine did not block SARS-CoV-2 entry in TMPRSS2-expressing cells. Novel inhibitors have been developed against TMPRSS2-mediated spike activation at the plasma membrane and have been shown to be effective against SARS-CoV-2 entry in both human lung epithelial cells and murine infection models [115,116,117]. 

However, newly emerged spike Omicron subvariants became less dependent on TMPRSS2-mediated spike activation at the plasma membrane and are insensitive to original inhibitors such as camostat mesylate [97,99,118]. While ACE2 remains an obligatory factor, a change in Omicron protease tropism has resulted in decreased replication in the lung and intestinal cultures, but a similar rate of replication in the nasal epithelia when compared to the Delta variant [98,99,119,120]. A change in spike protease tropism has also led to decreased lung pathogenesis in both human and animal models. It is likely that Omicron subvariants evolved towards ACE2 binding of the nasal epithelia, in order to favor air transmission through the upper respiratory tract, rather than TMPRSS2 usage in alveolar cells of the lower respiratory tract. Notably, cellular localization of TMPRSS2 on the plasma membrane is controversial, since past studies suggested that TMPRSS2 is mainly expressed in endosomes to promote IAV entry [121]. Mature TMPRSS2 has a C-terminus peptidase domain that requires an acidic environment to function optimally [122,123]. Hence, the relevance of TMPRSS2 for the activation of future SARS-CoV-2 Omicron subvariants remains to be explored. 

Other members of the TTSP family also promote SARS-CoV-2 spike-mediated entry in a context-dependent manner [124]. TMPRSS11D, also known as human airway trypsin-like protease (HAT), not only promotes SARS-CoV, but also SARS-CoV-2 viral entry and syncytium formation [70,125]. Moreover, SARS-CoV-2 spikes utilize both TMPRSS2 and TMPRSS4 to gain viral entry into human enterocytes of the proximal digestive tract [126], suggesting the synergic activation of spikes at S2′ by multiple proteases. An overexpression study has also shown TMPRSS13 is involved in the proteolytic activation of the spike protein [125]. However, the utilization of TMPRSS11D and TMPRSS13 is also impaired in Omicron subvariants [94]. Lastly, overexpression of the subtilisin-like furin has been shown to increase the S2′ cleavage and activate the spike-mediated membrane fusion even in the absence or presence of ACE2 [111], suggesting that furin may play additional roles during the viral entry process in addition to the S1/S2 autocleavage.

Cathepsin L has been implicated in the activation of the spikes of SARS-CoV and SARS-CoV-2 in the target cell endosomes [127,128]. Pharmacological inhibition of cathepsin activities via chloroquine [112], E64D, ammonium chloride and bafilomycin A1, or genetically interfering upstream clathrin-mediated endocytosis, blocks spike-mediated lysosomal entry in TMPRSS2-deficient cells [129,130,131]. These inhibitors are also effective against the Omicron subvariants, highlighting the importance of SARS-CoV-2 endosomal entry in the current global spread. It is still unclear whether cathepsin L is directly involved in the processing of the S2′ cleavage site or indirectly facilitates its cleavage via other lysosomal hydrolases, since no predicted cathepsin L cleavage sites are located near to the spike fusion peptide [132,133]. In addition, the maturation of various cathepsins requires stepwise transport, trafficking, and luminal pH of endolysosomes. For instance, recently identified LYSET/TMEM251 regulates the maturation of multiple cathepsin precursors and is required for the endocytic entry of SARS-CoV-2 [134]. Lastly, the coronavirus spike is not spontaneously cleaved or activated by cathepsins inside lysosomes during viral assembly, since β-coronaviruses utilize deacidified lysosomes to egress from infected cells [135]; this process can be facilitated by other SARS-CoV-2 components, such as ORF3a-mediated exocytosis [136].

Secreted and soluble protease may also play a role in the activation of the spike protein and exacerbating host pathogenesis. For instance, matrix metalloproteases (MMPs), such as ADAM10 and ADAM17, cooperatively cleave SARS-CoV-2 spikes into S2′ at the cell surface in TMPRSS2-deficient cells [137,138,139,140,141], and can be broadly inhibited by marimastat, batimastat, and TAPI-1. An early study showed that MMP inhibitors had no effect on spike S1/S2 cleavage [12], suggesting that MMPs may play a role during the activation of the spikes. However, Omicron subvariants not only reduced TMPRSS2 usage, but also reduced the metalloproteinases usage at the plasma membrane, since these inhibitors did not block Omicron BA.1 and BA.2 entry via endocytosis [142]. Certain proteases can be contributed by infiltrating innate immune cells, such as the neutrophil elastase (ELANE), which specifically cleaves the threonine 795 residue of the SARS-CoV spike upstream of the S2′ (R797) cleavage site [143]. Interestingly, this elastase cleavage site is not conserved in the current SARS-CoV-2 spike. Inhibiting the elastase activity also improves the adjuvanticity of the spike vaccine in vivo, suggesting that elastase may modulate spike antigen processing in the absence of membrane fusion [144]. Lastly, coagulation factors in the patient serum, such as factor Xa and thrombin, can directly cleave spikes at both S1/S2 and S2′ cleavage sites [145,146]; these factors may increase SARS-CoV-2 infection and exacerbate host pathology.

Spike substitutions and deletions not only profoundly impact viral transmission and immune evasion, but also critically determine host receptor and protease tropism. It is still unclear what protease is primarily involved in the activation of Omicron subvariants; nor inhibitors that selectively and efficiently block the endocytic route of SARS-CoV-2 entry. More systemic, physiological and context-dependent approaches on spike-activating proteases are warranted, in order to fully understand the mechanism of SARS-CoV-2 spike-mediated entry. Viral entry is the first and crucial step during the viral life cycle. Targeting viral entry could block the subsequent steps of virus infection and inhibit downstream viral replication and host cell pathology. 

## 5. Cellular Innate Immune Responses to Spike-Mediated Entry

The host innate immune response serves as the initial defense against a broad range of pathogens and shapes an essential adaptive immune response to clear viral pathogens [147]. At the resting state, the host innate immune response is assumed to be minimal to prevent spontaneous damage to tissue and organs. Upon sensing of the pathogen- or damage-associated molecular patterns (PAMPs and DAMPs), tissue residents and infiltrating immune cells ensure that a robust pro-inflammatory response is rapidly mounted. For a viral infection, the outcome of this innate immune response is to effectively break the cycle of viral entry and replication. Ultimately, host cells achieve an antiviral state that controls the infected tissues and prevents further infection. It is important to understand how the human innate immune response targets the SARS-CoV-2 spike-mediated entry and subsequent pathogenesis. 

**Figure 3:**
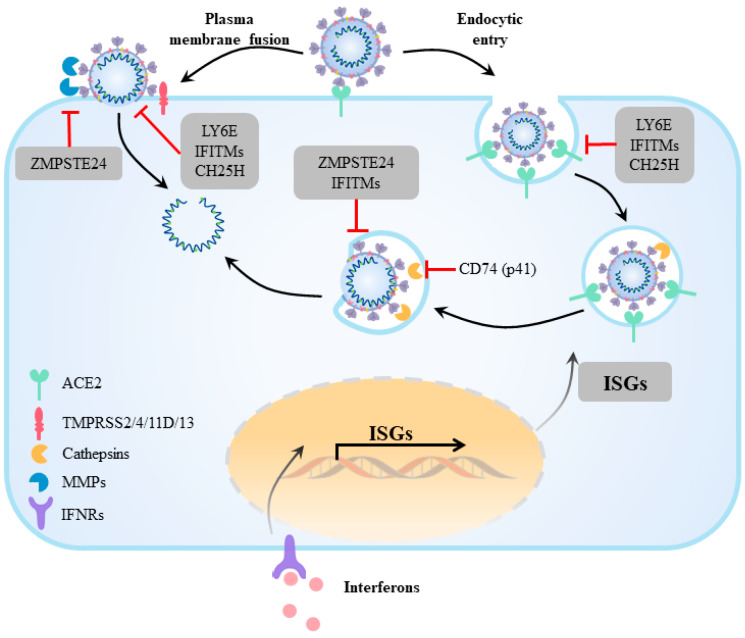
Host-derived antiviral effectors targeting the SARS-CoV-2 spike-mediated entry. SARS-CoV-2 virions can enter cells through membrane fusion at the plasma membrane or undergo endocytosis. Host-derived ISGs play distinct roles in various subcellular localizations to inhibit virus-mediated membrane fusion, proteases activation, or internalization.

Interferon-stimulated genes (ISGs) are the cellular intrinsic defense against viral infection. Interferon-induced transmembrane proteins (IFITMs) are members of the membrane-bound ISGs (Figure 3), comprised of IFITM1, IFITM2, IFITM3, IFITM5, and IFITM10. IFITMs are type IV transmembrane proteins with amphiphilic helixes interacting with the inner membrane, and can be post-translationally modified via palmitoylation, phosphorylation, and ubiquitination [148]. IFITMs are distributed in the early and late endosomes or plasma membrane to directly modulate the membrane functions of the viral fusion proteins. They have been shown in most cases to inhibit coronavirus infections, but sometimes they enhance infections. In an overexpression screening of functional ISGs, human IFITM2 and IFITM3 inhibited SARS-CoV-2 spike pseudotype infection [149], while overexpression of IFITM1 reduces spike-mediated cell–cell fusion [72]. In a separate study, the overexpression of IFITM3 did not restrict but enhanced the spike-mediated infection in TMPRSS2-expressing cells [150], suggesting that altered usage of host protease or fusion at the plasma membrane subverts the effect of host IFITMs. Mechanistically, IFITMs can block enveloped virus entry by blocking the fusion step in the semi-fusion stage at the target cell plasma membrane or endosomes. At the fusion pore stage, IFITMs have been proposed to reduce the fluidity of the plasma membrane or expand the curvature of the outer membrane lobule (Table 2).

Some independent evidence supports that the spike of SARS-CoV-2 interacts with human IFITMs, especially with IFITM2. IFITM2 interacts with SARS-CoV-2 spike proteins on the cell surface, mainly promoting subsequent virus–cell fusion in the early endosome [151]. It has been reported that overexpression of IFITM2 in HEK293T cells prevents spike-mediated membrane fusion as well as authentic SARS-CoV-2 viral entry, but the opposite effect has been observed in human lung epithelial cells endogenously expressing IFITM2. Silencing of all three IFITM proteins can reduce SARS-CoV-2 infection, while IFITM2 showed the strongest effects. IFITM may affect susceptibility to infection and the infectiousness of SARS-CoV-2 progeny virions [151]. Even though the IFITMs were reported to affect cholesterol hemostasis, the antiviral mechanism of IFITM3 was not due to regulating cholesterol synthesis/transport [148]. Human IFITM3 restricts spike-mediated SARS-CoV-2 entry at the endosomes and requires its YxxΦ endocytic sorting motif for its antiviral activity [150]. A YxxΦ motif mutant led to an enrichment of IFITM3 at the plasma membrane, which lead to enhanced membrane fusion and syncytium formation mediated by the SARS-CoV-2 spike, a phenotype that could be associated with the increased pathology in humans [150]. Indeed, a human IFITM3 rs12552-C single nucleotide polymorphism (SNP) impairs the YxxΦ sorting motif and is strongly associated with SARS-CoV-2 patient severity and hospitalization in people under 60 years of age in Saudi Arabia [152]. The inhibitory effect of IFITM proteins is broad and could mechanistically involve membrane rigidity, curvature, and endosomal trafficking, instead of specific interactions with viral glycoproteins [150,153]. Further research is needed to fully clarify the mechanism of IFITM-mediated inhibition and enhancement of SARS-CoV-2 viral entry.

Cholesterol-25-hydroxylase (CH25H) is one of the intracellular ISGs, which synthesize 25-hydroxycholesterol (25HC) from an intracellular pool of cholesterol (Figure 3). 25HC has been previously shown to exhibit effective broad-spectrum antiviral activity. Clinical and in vivo studies have shown that 25HC was elevated in COVID-19 patients as well as in SARS-CoV-2-infected human ACE2 (hACE2)-transduced mice. There was a potent antiviral effect on SARS-CoV-2 infection induced via CH25H expression in vitro and in vivo [154]. Our previous work proved the antiviral activity and underlying mechanism of CH25H against human coronaviruses (Table 2). 25HC directly blocks membrane fusion, since it effectively reduces spike-mediated cell–cell fusion in the absence of other viral components [155]. Mechanistically, the ER-localized acyl-CoA: cholesterol acyltransferase (ACAT) can be activated by CH25H enzymatic product 25HC, leading to the depletion of accessible cholesterol from the plasma membrane and restriction of viral entry by inhibiting membrane fusion [155]. In addition, elevated 25HC accumulates in the late endosomes when binding to Niemann-Pick C1 (NPC1), resulting in the inhibition of SARS-CoV-2 spike protein-catalyzed membrane fusion by preventing cholesterol export [156]. 

The lymphocyte antigen 6 complex member E (LY6E) gene is a member of the lymph stromal cell membrane Ly6 superfamily. It is a glycosylphosphatidyl-inositol (GPI)-anchored cell surface protein, rich in lipid raft microdomains, which regulate cytoskeletal rearrangement and the membrane-dependent pathways, such as the endocytosis and signaling pathways. LY6E can effectively reduce cell infection caused by multiple coronaviruses, including SARS-CoV, SARS-CoV-2, MERS-CoV and mouse hepatitis virus (MHV) [157], but promote other human pathogenic viruses such as HIV and flaviviruses [158,159]. The effect of LY6E on different viruses largely depends on its GPI anchoring [157,160]. LY6E directly blocks SARS-CoV-2 spike-mediated membrane fusion [161] (Figure 3), and could rely on an interesting mechanism by regulating the size of the endosome [162]. The inhibitory effect of LY6E is less dependent on the expression of host proteases [157], such as TMPRSS2, and could potentially be effective against Omicron subvariants. Ly6e-deficient mice are highly susceptible to coronavirus infection and developed increased severity, accompanied by the loss of immune cells in the liver and spleen [161]. These observations made LY6E an antiviral factor of the spike-mediated entry. 

Cells express endogenous antiviral factors to inhibit proteolytic activation of spikes. For instance, activation of the class II major histocompatibility complex transactivator (CIITA) leads to the upregulation of CD74 p41. P41 is a unique short isoform of the CD74 that carries a functional thyroglobulin type-1 domain, which inhibits cysteine proteinases, such as cathepsins [163]. Interestingly, the lack of an N-terminal endoplasmic reticulum (ER) signal enables p41 to retain in the host endosomes and inhibit cathepsin-mediated entry of SARS-CoV-2. Due to changes in protease tropism, it is still unclear whether CD71 p41 play more important roles in curbing the ongoing Omicron spike-mediated entry. Zinc metallopeptidase STE24 (ZMPSTE24) is expressed as a partner of IFITMs to defend against entry of many enveloped viruses via an unknown mechanism [164,165]. Unlike most metalloproteases, the multi-membrane spanning ZMPSTE24 is a restricting factor of SARS-CoV-2 infection and syncytia formation at the plasma membrane; genetic interference of ZMPSTE24 expression resulted in enhanced spike-mediated pseudotype entry [166]. Expression of a catalytically dead ZMPSTE24 mutant is equally protective as the wildtype, suggesting that ZMPSTE24-mediated inhibition of the spike is independent of its catalytic function. Hence, the molecular mechanism of ZMPSTE24-mediated inhibition requires further investigation.

## 6. Inflammatory Processes beyond ISGs

Before the involvement of adaptive immune responses, timely innate immune responses are paramount for sensing and achieving the antiviral states in SARS-CoV-2 infected tissues. In target cells such as the lung epithelia, pattern recognition receptors (PRRs) are responsible for the initial recognition of SARS-CoV-2 components either extracellularly or intracellularly [167]. These include the detection of membrane-bound spikes and envelope proteins by toll-like receptors [168,169], or viral single-strand RNA by intracellular RIG-I and MAVS [170]. These cells then secrete soluble cytokines, chemokines, and hormones, aiming to evoke and propagate signaling events in either adjacent tissues or distant organs to prepare for possible infection. Consequentially, antiviral factors and ISGs are produced to prevent further infections, and the mechanisms of these host’s innate immune defense against SARS-CoV-2 are an area of intense research interest. 

In addition to ISGs and soluble factors, intracellular inflammasomes are intracellular innate immune sensors constitutively expressed in the respiratory epithelia and circulating immune cells. For instance, NLRP1 senses the SARS-CoV-2 dsRNA and cleavage by 3CL protease in human lung epithelial cells [171,172]; in turn, inflammasome activation then drives the secretion of proinflammatory cytokines such as IL-1β and IL-18. In contrast, innate immune cells without ACE2 expression can be also infected with the SARS-CoV-2 virus via Fcγ receptors [173,174], and is implicated in propagating the inflammatory responses in human lung tissues [175,176]. Another outcome of SARS-CoV-2-triggered inflammasome activation is the inflammatory form of cell death, namely pyroptosis [177]. During this process, human gasdermin D and E form membrane-permeable pores that not only limit the intracellular viral replication [172], but also ensure the release of DAMPs from infected lung epithelial cell and monocytes [173]. Spike-driven syncytium formation between pneumocytes is sufficient in triggering the cleavage of gasdermin E and pyroptosis [177]. Hence, these alternative innate immune processes present in SARS-CoV-2-targeted cells produce robust signals for bystander epithelial cells, as well as the infiltrating innate immune cells to relay the antiviral state. Interestingly, inflammasome-driven cytokine release is also crucial for vaccine-mediated protection [178], suggesting that pro-inflammatory mediators released during pyroptosis could orchestrate and fine-tune subsequent tissue-dependent adaptive immune responses. 

Lastly, antigen-independent immunity can be raised against SARS-CoV-2 spike-mediated entry. The Bacillus Calmette–Guérin (BCG) vaccine is a non-viral, live-attenuated vaccine for the bacteria *Mycobacterium tuberculosis*. Interestingly, it provides significant protection against the SARS-CoV-2 infection in a mouse model of authentic infection [179]. Moreover, the BCG has been demonstrated as a more potent vaccine adjuvant than alum [180]. Injection of the BCG vaccine is unlikely to instruct the generation of cross-reactive T cells or antibodies; yet, the short-term protection against SARS-CoV-2 infection has been observed in the animal model, and may be derived from the primed innate immune and tissue cells. A study of BCG-vaccinated health care workers has also shown a decreased seroprevalence of SARS-CoV-2 [181]. Although studies contradict the effectiveness of the BCG vaccine against SARS-CoV-2 [182,183], further assessment of BCG-mediated innate immune responses in humans are needed.

## 7. Conclusions

The SARS-CoV-2 virus will continue to evolve, and it is currently crucial to establish the fundaments of its biochemical modes of entry. From host receptor recognition, protease-mediated proteolytic processing, structural rearrangement, and fusion pore formation, each of these steps requires further investigation in physiologically relevant and context-dependent models. Regardless of the virion or cellular spikes, molecular mechanisms underlying the activation mode and membrane fusion are still elusive. Understanding the converging proteolytic processing of spikes ensures rapid identification, screening, and application of novel protease and fusion inhibitors. There is also a continued need for the identification of new host factors that regulate SARS-CoV-2 infection, especially for emerging new VOC. 

Host-derived innate immune factors are likely to act as broad-spectrum, endogenous inhibitors of spike-mediated viral entry. While there have been many successful works in this field, we should still notice that some factors exhibit contradictory mechanisms and phenotypes in different models. Overcoming this problem may require the utilization of 3D and physiologically relevant models such as the lung organoid and air–liquid interface culture of the human airway epithelia. Further, we should also pay attention to the differences in the ISGs’ antiviral effects against ancestral strains and newly emerged variants. Overall, these findings further our understanding of the host–virus arms race during viral entry and provide wider clues for novel antiviral therapeutics against emerging and re-emerging infectious diseases. 

## Figures and Tables

**Table 1:** SARS-CoV-2 converging mutations and phenotypes on S1/S2 cleavage and modes of spike-mediated entry.

Phenotypes	Wuhan-Hu-1, Alpha, Beta, Delta	Omicron and Subvariants BA.1, BA.2 … BQ.1.1 and XBB.1.5
Conserved mutations	D614G	D614G, H655Y, Q954H and N969K
S1/S2 cleavage	Strong	Reduced
Plasma membrane entry	Yes	No
Endocytic entry	Yes	Yes
TMPRSS2 Usage	Yes	No
Predominantly targeted tissues	Nasal cavity, lung	Nasal cavity
Risk of lung pathogenicity	High	Low

**Table 2:** Characteristics of ISGs regulating human coronavirus entry.

ISG Name	Subcellular Localization	Affect Step of Virus Entry	Molecular Mechanisms	Other Coronaviruses
IFITMs ^1^	Early and late endosome membranes, plasma membrane	Semi-fusion stage at the target cell plasma membrane or endosomes	Reduce the fluidity of the plasma membrane or expanding the curvature of the outer membrane lobule	hCoV-229E, SARS-CoV, MERS-CoV
CH25H	Endoplasmic reticulum membrane, plasma membrane, cytosol	Reduces spike-mediated cell-cell fusion	Converts cholesterol to 25HC, leading the depletion of accessible cholesterol from the plasma membrane	SARS-CoV, MERS-CoV, HCoV-229E, HCoV-OC43
LY6E	Glycosylphosphatidyl-inositol (GPI)-anchored cell surface	Directly blocks membrane fusion	Directly blocks membrane fusion, depending on its GPI anchoring, being less dependent on the expression of host proteases, such as TMPRSS2.	SARS-CoV, SARS-CoV-2, MERS-CoV, MHV
CD74 ^2^	Plasma membrane	Inhibits cathepsin-mediated entry	Functional thyroglobulin type-1 domain inhibits cysteine proteinases	hCoV-229E, hCoV-OC43, SARS-CoV, MERS-CoV
ZMPSTE24	Plasma membrane, endomembranes	Bingding, membrane fusion	Mediates cleavage and shedding of the ACE2 ectodomain interacts with IFITMs	hCoV-229E, hCoV-OC43, SARS-CoV, MERS-CoV

^1^ It mainly includes IFITM1, IFITM2, and IFITM3. ^2^ It basically means p41 isoform.

## Data Availability

Not applicable.
